# Quantification of aortic regurgitation and stroke volume by CMR - variation due to slice plane position. It matters where you measure!

**DOI:** 10.1186/1532-429X-13-S1-P203

**Published:** 2011-02-02

**Authors:** Christian R Hamilton-Craig, Peter J Cawley, Abhishek Chaturvedi, Gregory J Wilson, William Kerwin, Catherine M Otto, Jeffrey H Maki

**Affiliations:** 1 University of Queensland, Brisbane, Australia; 2 University of Washington, Seattle, WA, USA; 3 Philips Healthcare, Cleveland, OH, USA

## Purpose

Aortic regurgitation by CMR has been assessed in various locations, including sinus of valsalva (SOV), sinotubular junction (STJ), and ascending aorta (ASC). Variability in obtained measurements and interchangeability of these locations and methods in patients with varying valvular disease severity is unknown. We sought to determine the most appropriate aortic level for accurate phase contrast quantitative (Q) flow measurement of forward and backward flow and calculation of Qp:Qs in patients with valvular heart disease.

## Methods and materials

57 patients with valvular disease (31 aortic regurgitation (AR), 25 mitral regurgitation (MR), 1 both) were imaged at 1.5T using Q flow at 3 ascending aorta locations: sinus of valsalva (SOV), sinotubular junction (STJ), tubular ascending aorta 1 cm above STJ (ASC). Pulmonary artery (PA) Q flow was performed at two adjacent locations 1 cm apart. No patient had intracardiac shunt. Blinded analysis was performed by two expert readers with SCMR level 2 and 3 experience. Net stroke volume (SV), forward volume (FV) and backward volume (BV) were measured and analyzed by Student t-test**.**

## Results

**Figure 1:**
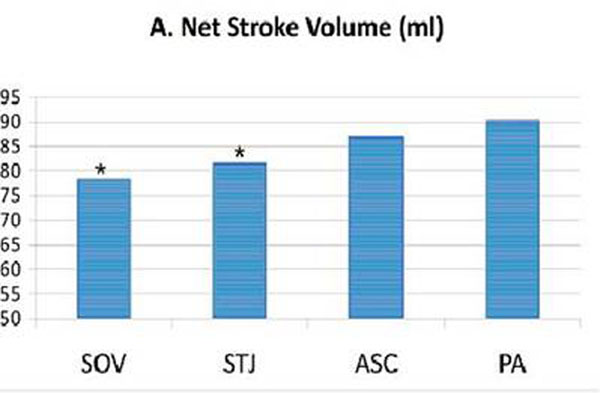
Net Stroke Volume at four sites of breath-held Qflow acquisition (* =*p<0.001*)

No significant SV/FV/BV difference was seen between the two PA locations, and this SV was deemed the best representation of truth (Qp). The best aortic (Qs) correlation with Qp occurred at the ASC (87.1 ± 17 ml, p = 0.82), with a significant drop in SV moving proximally to the STJ (81.7 ± 20.0 ml, p<0.001) and SOV (78.5 ± 18ml p<0.001) (Figure 1).

BV decreased significantly as acquisition moved further from the aortic valve (SOV:STJ -6%, SOV:ASC -18%, both p<0.001). Correspondingly, regurgitant fraction decreased significantly moving distally; SOV 23 ± 19%, STJ 20.5 ± 19% (p< 0.001), ASC 17.7 ± 17% (p< 0.001). FV trended higher distally, particularly for patients with significant AR.

## Conclusion

Aortic Qflow is influenced by slice plane, with FV and BV affected differently. Total net SV (summed throughout the cardiac cycle and therefore not influenced by aortic distension and through-plane motion) is most accurately measured in the ASC. This is relevant when quantitating Qp:Qs. Aortic regurgitation (BV and RF) is most sensitively detected proximally, with significant reductions in detected regurgitant flow when measured distally. Inaccuracy of FV when detected proximally appears to be due to phase errors, particularly in AR patients with turbulence. A complete exam should include phase contrast measurements at both the ASC and SOV.

